# Determining the seasonality of respiratory syncytial virus in Slovenia

**DOI:** 10.1111/irv.12779

**Published:** 2020-07-12

**Authors:** Eva Grilc, Katarina Prosenc Trilar, Jaro Lajovic, Maja Sočan

**Affiliations:** ^1^ National Institute of Public Health Ljubljana Slovenia; ^2^ National Laboratory of Health, Environment and Food Ljubljana Slovenia; ^3^ Ro Sigma Ljubljana Slovenia

**Keywords:** MEM, RSV, season threshold, seasonality, surveillance

## Abstract

**Background:**

In Slovenia, the respiratory syncytial virus (RSV) surveillance is based on national laboratory data. The weeks with more than 10% of samples tested positive compose RSV epidemic season. The use of real‐time multiplex PCR, which identifies other respiratory pathogens in parallel with RSV, caused more testing but the percentage of RSV positives lowered. The 10% threshold was reached with delay, which raised concern about its suitability for defining RSV seasonality.

**Methods:**

To describe the seasonality of RSV, the onset, offset and duration of the RSV epidemic season across 10 years (from week 40, 2008/2009 to week 39, 2017/2018), four calculative methods were deployed including moving epidemic method, MEM, and epidemiological parameters were compared.

**Results:**

In 10 years, 10 969 (12%) out of 90 264 samples tested positive for RSV. The number of tested samples increased remarkably from the first to last season with a drop in the percentage of positive samples from 23% to 10%. The onset of RSV epidemic varied considerably regardless of the calculative method used (from 10 to 13 weeks). The unevenness in the RSV epidemic season end was also observed. The average duration of RSV epidemic season was the shortest when moving epidemic method has been used (15.7 weeks) and longest with ≥3% method (22.9 weeks).

**Conclusion:**

The ≥3% calculative method could be used as an early warning of the RSV season. However, ≥7% calculative method was found to be reliable enough to define the epidemiological parameters of an ongoing season and to support public health response. The potential of the moving epidemic method should be further explored.

## 1 INTRODUCTION

Respiratory syncytial virus (RSV) is a common cause of childhood acute respiratory infections and a major cause of hospital admissions in young children.^1^ The public health importance of RSV is due, at least in low‐income settings, to its high morbidity and mortality in young children.^2^

The disease burden among the elderly population and people with chronic diseases like COPD is substantial as well and may be similar to that of seasonal influenza A virus infection in some seasons.

It has been estimated recently that RSV causes 33.1 million episodes of RSV‐associated lower respiratory tract infections globally, which results in about 3.2 million hospital admissions, and approximately 60 000 in‐hospital deaths in children younger than 5 years in developing countries.^3^ The contribution of adult RSV infections to the utilisation of advanced healthcare resources, morbidity and mortality has become increasingly clear in recent years.^4^, ^5^ Respiratory syncytial virus season results in a substantial burden on healthcare services.^6^, ^7^ RSV infections have significant health, financial and social impact in both high‐ and low‐income countries.^8^

In regions with a temperate climate, RSV starts circulating in the autumn, peaks in winter and ends in spring.^9^, ^10^, ^11^, ^12^ The onset and offset of RSV season are variable from year to year.^13^ The variability may depend on temperature, humidity, precipitation and other environmental and social drivers of seasonality.^14^ Furthermore, variation in epidemic size and the timing of the RSV season have been linked to subtype predominance. According to surveillance data from Beijing, China, the season onset and peak in RSV A prevailing years occurred 3‐5 weeks earlier and duration was 6 weeks longer than those observed in RSV B prevailing years.^15^ On the other hand, European studies have not confirmed that.

There are some studies available that studied the clinical impact of viral factors during RSV infection, and some studies have reported that different subtypes and genotypes lead to different disease severity,^15^, ^16^, ^17^, ^18^ while others have shown that they have equivalent severity.^19^, ^20^, ^21^

A global study that included data from Europe, America, South Africa, Oceania and Asia on RSV did not analyse the circulation of the different RSV subtypes and its impact on disease severity, because almost no surveillance network differentiates between the different genotypes. The literature is based on small sample studies carried out for short periods, preventing a global review of the subject.^22^

Recognising the beginning and end of RSV season is important from a public health perspective and for healthcare providers.^16^

Knowing the start and end of the RSV season in any given locality is important to healthcare providers and public health officials who use RSV seasonality data to guide diagnostic testing, information for parents of vulnerable children and the timing of RSV immunoprophylaxis for children at high risk of severe respiratory infection. This highlights the need for updated and precise epidemiological data on infection with RSV to optimise the start and end of prevention with palivizumab and to inform clinical care and in the near future on vaccine use as well.

Recommendations on the timing and duration of palivizumab prophylaxis rely on national/regional surveillance data.^17^ Knowledge of the seasonal trends of RSV infections and a better understanding of what is driving these seasonal patterns provides many benefits, including health system preparedness for increased pressure on primary care and hospitals.^10^

In Slovenia, from 2006 onward, children with serious congenital heart disease, chronic pulmonary disease or born prematurely (before the 29th week of gestation) are, in their first year of life, given human monoclonal antibodies against RSV (palivizumab) during the RSV season. The beginning, top and end of RSV circulation are detectable if only the respiratory tract samples are collected from patients with acute respiratory infections and tested on RSV since the course of RSV infection is clinically indistinguishable from other respiratory infections.^16^ There is no generally accepted method to define the start and the end of the RSV epidemic season. Different methods are in use.^9^, ^10^, ^11^, ^18^, ^19^ In Slovenia, laboratory‐based RSV surveillance was established in the year 2006. The start and the end of the RSV epidemic season were set on a 10% threshold—the weeks when more than 10% of samples tested positive composed the RSV epidemic season. The currently used 10% threshold was set according to CDC (Centre for Disease Control) guidelines.^9^ In the USA, the RSV season was defined by consecutive weeks when RSV antigen‐based tests exceeded 10% positivity. However, since 2008, laboratories in the USA have shifted away from antigen‐based RSV testing, and since 2014, the majority of tests and RSV detections among consistently reporting laboratories are determined by PCR.

The situation in Slovenia regarding testing practice has changed as well.

The number of samples tested has increased, while real‐time multiplex PCR has been used intending to identify other respiratory pathogens concomitantly—notably influenza viruses. The number of RSV positives increased to some extent but the percentage of tests positive for RSV lowered. The 10% threshold was reached with delay, which raised concerns about its suitability for epidemiological purposes. On the other hand, in similar study done in the Netherlands ^19^ percentage of RSV positives increased. However, situation in Slovenia and the Netherlands cannot be compared easily. Data from the Netherlands are based on general practitioner(s) data and hospital data. Data in Slovenia are based on hospital data. And more selective testing on RSV in the Netherlands ordered by primary practitioners is consequence of informed testing and awareness of RSV problem.

In our study, we tested methods for a more precise determination of RSV seasonality in Slovenia. We were interested in how the start and the end of RSV season are affected by a lower positivity rate with different calculation methods. The objective of the study was to compare epidemiological parameters calculated with each method and to evaluate suitability and usefulness for public health objectives of RSV surveillance. Reliable RSV surveillance and epidemiological data can moreover provide a baseline to assess possible effects of RSV vaccine in near future.^23^

## 2 METHODS

### 2.1 Data source

A respiratory syncytial virus surveillance programme was initiated in Slovenia in 2006.^20^ The National Institute of Public Health invited public health and clinical laboratories to participate voluntarily in the programme. Each participating laboratory was asked to report on a weekly basis the number of patients tested for RSV and the number of positive/negative results.

Sampling instructions were given to laboratories and physicians and did not change over the years. But mostly samples were taken for diagnostic purposes in physicians sole discretion. In the first years when tests were still separated, physicians recorded RSV testing when they suspected for RSV infection. The reason for less selective testing in following years might be the availability and affordable prices of multiplex PCR kits (respiratory panel).

Currently, there are nine laboratories testing for RSV in Slovenia—a laboratory for respiratory viruses at the Institute for Microbiology and Immunology, Medical Faculty, serving the largest teaching hospital in the capital, two regional hospital laboratories and six public health laboratories, which provide laboratory support for regional hospitals and primary care in local environments. Public health laboratories are organisationally part of the same institutional setting (National Laboratory of Health, Environment and Food—NLHEF) at different locations. Seven laboratories (out of nine) provide regularly aggregated weekly data on RSV testing.

One hospital laboratory declined to participate. One laboratory, namely the Laboratory for Public Health Virology (LPHV), NLHEF, is not included in the RSV surveillance programme. The LPHV serves as an NIC (National Influenza Centre) and receives respiratory samples for influenza surveillance programmes only. Healthcare providers participating in influenza surveillance programmes were instructed to take samples from patients with influenza‐like illness (ILI) according to the definition. Though LPHV tests (in the frame of an influenza surveillance programme) for RSV 100% of samples of patients with ILI, the proportion of positive samples is still low as programme targets at influenza virus. LPHV screens for RSV subgroup A (RSV A) and B (RSV B) all positive samples from 2011 on. Consequently, LPHV serves as a source of RSV subgroup seasonal predominance over the last seven seasons. Dominance was determined on the basis of a 60/40 rule used in influenza surveillance.^21^

Reporting laboratories receive 100% of their samples from hospital settings. The system covers 4% of the Slovenian population which is a simple representative sample of all population in Slovenia.

All reporting laboratories are using real‐time PCR for RSV diagnostics. At the beginning of the programme, before the year 2001, a few laboratories used antigen detection tests (direct immunofluorescence or immunoassays), but these tests were omitted in the following seasons and replaced by real‐time PCR tests after the year 2001. Most often, laboratories perform RSV testing simultaneously with testing for influenza A and B viruses using commercial multiplex PCR systems. Differentiation of RSV subtypes A and B was achieved by real‐time PCR.^24^

The National Institute of Public Health assembles RSV laboratory surveillance data and makes the data publicly available every Friday on their website.^25^

### 2.2 Data analysis

To describe the seasonality of RSV, the onset, offset and duration of the RSV epidemic season across ten years (from week 40 in 2008/2009 to week 39 in 2017/2018), four methods were deployed. The definitions used to determine RSV epidemic seasons were the following:

1. The onset week was defined as the first of two consecutive weeks when the weekly percentage of specimens testing positive for RSV was ≥3%,^7^ ≥5%, ≥ 7% or ≥10%^26^ with not less than 20 specimens tested per week. The offset week was the last of two consecutive weeks when the weekly percentage of specimens testing positive for RSV was ≥3%,^7^ ≥5%, ≥7% or ≥10%.^27^ No gap week was allowed.
2. The weeks when RSV detections exceeded 1.2% of total RSV‐positive specimens per season with one gap week allowed.^11^
3. The weeks with ≥60% of each year's average weekly number of laboratory detections of RSV‐positive specimen.^19^
4. Using moving epidemic method (MEM)^20^ with the weekly percentage of samples with acute respiratory infections.

The MEM input is based on weekly percentages of positive specimens with respiratory syncytial virus among all clinically diagnosed cases with acute respiratory infections.

The MEM basically consists of 3 steps: (a) estimation of the epidemic period length (for each season separately, determined as the minimum number of consecutive weeks with the maximum accumulated percentage rates), splitting the season in a pre‐epidemic, an epidemic and a post‐epidemic period; (b) calculation of epidemic threshold (determined as the upper limit of the 95% one‐sided CI of 30 highest pre‐epidemic weekly rates); and (c) estimation of medium, high and very high‐intensity thresholds (determined as the upper limits of the 40%, 90% and 97.5% one‐sided CIs of the geometric mean of the 30 highest epidemic weekly rates).^26^ The advantage of the MEM is that it weekly monitors the intensity level of the infections with respiratory syncytial virus. We have used weekly percentage of specimens which tested positive as the MEM input.

Estimations by the MEM were performed using its R Language implementation. The MEM epidemic threshold calculated for the overall 12‐year period of our historical data was 25.46.

The peak week of the RSV epidemic season was defined: the week with the highest percentage of positive samples (for method 1), the week with the highest percentage of total RSV‐positive specimens (for method 2) and the week with the highest number of weekly RSV detections (for method 3). For the moving epidemic method, the peak week of the RSV epidemic was determined as the week with the maximum rate within the epidemic period (which was estimated as mentioned above).

We calculated for each method the percentage of all RSV detections that occurred within the RSV epidemic season according to the definition.

## 3 RESULTS

**Figure 1:**
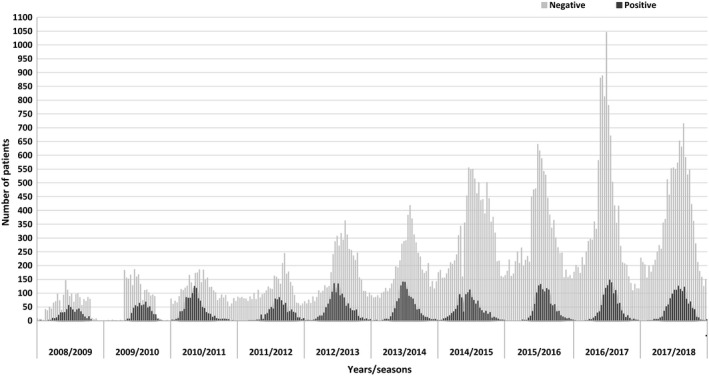
Number of samples tested negative or positive for RSV from Week 40/2008 to Week 39/2018, Slovenia

In a 10‐year period (from week 40/2008 to week 39/2018), 90 264 samples were tested for RSV and 10 969 (12%) were found to be positive. The number of tested samples increased from 2342 in the first season to 13 583 in the last season. The number of samples positive for RSV increased from 539 to 1384 in the ten‐year period, but the percentage of RSV positives diminished from 23% in season 2008/2009 to 10% in season 2017/2018 (Figure 1).

After the 2010/11 season, the type of RSV virus was confirmed as well. In the six seasons that followed: 2011/12, 2012/13, 2013/14, 2016/17 and 2017/18, the RSV A or RSV B virus were predominant. During the 2015/16 season, the percentage of virus RSV A and RSV B was almost the same (Table 1).

**Table 1:** RSV A and RSV B seasons from 2011 to 2018

Seasons	Number of tests	Number of RSV positive	% of RSV positive	% positive RSV A	% positive RSV B
2011/2012	1478	109	7.37	73.39	26.61
2012/2013	2516	336	13.35	95.24	4.76
2013/2014	2208	171	7.74	26.90	73.10
2014/2015	3100	196	6.32	21.94	78.06
2015/2016	2528	223	8.82	48.60	51.40
2016/2017	2711	130	4.80	92.04	7.96
2017/2018	2309	214	9.27	30.84	69.16

**Figure 2:**
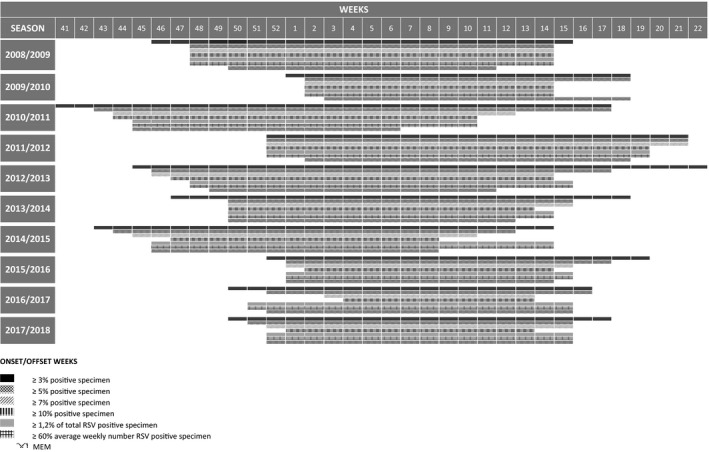
The onset and offset weeks of RSV seasons according to different calculative methods

The onset of RSV epidemic varied considerably regardless of the calculative method used. Using a traditional 10% threshold, the RSV epidemic season started as early as in Week 44 (season 2010/2011) or as late as in Week 4 (season 2016/2017) (Figure 2). The earliest start and the latest start of the RSV epidemic season were 13 weeks (≥10% and ≥3% method), 12 weeks (≥7% method), 11 weeks (≥5% method and MEM) and 10 weeks (≥1.2% and ≥60% method) apart. We observed the different RSV epidemic season offset. The minimum variability of the offset was 9 weeks using ≥3% method and a maximum of 13 weeks with MEM (Figure 2). There was a complete consistency in the start and the end of the season between ≥1.2% and ≥60% methods.

Respiratory syncytial virus epidemic season variations are observed every year, and there are also variations due to the calculation methods used.

The average duration of RSV epidemic season was the shortest when MEM has been used (15.7 weeks). The average season duration using different percentage thresholds was 22.9, 19.6, 17.5 and 15.7 weeks for ≥3%, ≥5%, ≥7% or ≥10%, respectively. The average duration of ten RSV epidemic seasons using predefined ≥1.2% and ≥60% threshold methods was 17.7 weeks.

The difference in RSV epidemic season length was reflected in the percentage of positive samples captured between the start and the end of the season (Table 1). The ≥3% calculative method captured from 97.4% to 99.6% (on average 97.8%) positive samples, and MEM from 77.2% to 94.9% (on average 89.0%) positive samples.

Respiratory syncytial virus A and RSV B subtypes were dominant in 3 seasons, respectively. In one season, according to the 60/40 rule, both subtypes co‐dominated.

Peak weeks of each RSV epidemic season are shown in Figure 2. In five seasons, the peak has been reached at the same time or one week apart whatever method used. In five seasons, the difference was more pronounced (from 2 to 5 weeks).

## 4 DISCUSSION

In the study, we used different calculative approaches to describe RSV activity in Slovenia.

Two methods out of four are retrospective (the ≥1.2% or ≥60% method). The epidemiological parameters can be calculated after the season is over. Methods using pre‐set ≥ 3%, ≥5%, ≥7% and ≥10% threshold enable evaluation of the season in real time (Table 2). The MEM is a prospective method. It models historical weekly rates of respiratory syncytial virus activity from past seasons to determine and quantify the expected activity levels of the respiratory syncytial virus season under surveillance. The main purpose of the latter method is that it calculates an epidemic threshold that serves as an alert signal for the upcoming epidemic.^27^

**Table 2:** Percentage of RSV captured during season

Season	S/O	Number of tested	poz RSV	% poz RSV	poz RSVA	% pozRSVA	poz RSVB	% pozRSVB	untyped	% untyped	% pozRSV type A	% pozRSV type B
2011‐12	Sentinel	225	18	8.00	5	27.78	13	72.22	0	0.00	27.78	72.22
	Others	1253	91	7.26	75	82.42	16	17.58	0	0.00	82.42	17.58
	Skupaj	1478	109	7.37	80	73.39	29	26.61	0	0.00	73.39	26.61
2012‐13	Sentinel	611	49	8.02	43	87.76	6	12.24	0	0.00	87.76	12.24
	Others	1905	287	15.07	277	96.52	10	3.48	0	0.00	96.52	3.48
	Skupaj	2516	336	13.35	320	95.24	16	4.76	0	0.00	95.24	4.76
2013‐14	Sentinel	578	42	7.27	23	54.76	19	45.24	0	0.00	54.76	45.24
	Others	1630	129	7.91	23	17.83	106	82.17	0	0.00	17.83	82.17
	Skupaj	2208	171	7.74	46	26.90	125	73.10	0	0.00	26.90	73.10
2014‐15	Sentinel	836	61	7.30	13	21.31	48	78.69	0	0.00	21.31	78.69
	Others	2264	135	5.96	30	22.22	105	77.78	0	0.00	22.22	77.78
	Skupaj	3100	196	6.32	43	21.94	153	78.06	0	0.00	21.94	78.06
2015‐16	Sentinel	536	42	7.84	22	52.38	16	38.10	4	9.52	57.89	42.11
	Others	1992	181	9.09	82	45.30	94	51.93	5	2.76	46.59	53.41
	Skupaj	2528	223	8.82	104	46.64	110	49.33	9	4.04	48.60	51.40
2016‐17	Sentinel	604	30	4.97	24	80.00	3	10.00	3	10.00	88.89	11.11
	Others	2107	100	4.75	80	80.00	6	6.00	14	14.00	93.02	6.98
	Skupaj	2711	130	4.80	104	80.00	9	6.92	17	13.08	92.04	7.96
2017‐18	Sentinel	262	61	23.28	22	36.07	39	63.93	0	0.00	36.07	63.93
	Others	2047	153	7.47	44	28.76	109	71.24	0	0.00	28.76	71.24
	Skupaj	2309	214	9.27	66	30.84	148	69.16	0	0.00	30.84	69.16

Ten years of data derived from clinical laboratories testing predominantly hospitalised patients were used to investigate the onset, offset, duration and peak of RSV season. In this time frame, the number of RSV tests performed increased more than fivefold with a drop in positivity rate. However, considering the fact that MEM threshold(s) are calculated over several seasons based on cumulative sequential time data, it would seem that this method is less sensitive to such trends.^28^ The assessment of national surveillance data for RSV in the Netherlands showed that, within sentinel general practice (GP) data, the percentage of RSV positives per season increased significantly over the 12 seasons.^18^ One possible explanation given by the authors is that this increase is a consequence of higher RSV awareness, resulting in patient samples being taken more selectively at the GPs.^19^ We assume that the decline in positivity rate observed in a laboratory‐based RSV surveillance system in Slovenia is a result of a less selective testing approach or even that samples were taken to confirm influenza virus using a multi‐pathogen panel. Therefore, our results cannot be compared to the results of the study in the Netherlands. The Dutch sentinel is based on GPs who do the informed and special testing on RSV. Furthermore, in Slovenia, laboratory testing is carried out in hospital settings where the primary aim is to test on influenza and the secondary on RSV.

We do not have data on the age distribution of the patients tested for RSV. It would be interesting to analyse whether there has been any change in the age distribution of patients tested for RSV recently. In case, more middle‐aged and elderly were tested, as this might support the hypothesis that influenza‐like illness was the main reason to perform the test in many cases and a negative result for RSV was an expected outcome. In order to understand the dynamics of testing better, it might prove useful to collect demographic data from routinely tested patients. Midgley et al^9^ analysed the impact of increasingly widespread use of multi‐pathogen PCR panels on RSV positivity rate. Compared to antigen‐based assays targeting RSV only, multiplex PCR aims to identify a set of viruses causing acute respiratory infection. The weekly RSV positivity rate was notably lower for PCR‐based reports than for antigen‐based reports.^9^

Slovenian data showed that the onset and offset of RSV epidemic season varied considerably from season to season no matter what calculative method used. There was practically complete concordance in the onset week through the 10‐year period between ≥1.2% and ≥60% method. The onset week defined with traditional ≥10% was aligned with the above‐mentioned two methods for four seasons, differed for one week in the other four seasons and deviated (being late for 2 and 5 weeks in season 2015/2016 and season 2016/2017, respectively). As expected, ≥3% method gave the earliest beginning of the season. The onset median was 50.5‐51 weeks for all methods, except for ≥3% method (median 48.5 weeks). As the median start week for RSV in Europe was found to be Week 49 and Week 51 in the USA, which shows good accordance with data from comparable climatic environments.^10^, ^13^ In areas with a temperate climate, annual patterns of RSV activity were strongly associated with the meteorological conditions.^28^ The mean temperature and atmospheric pressure were the main factors that correlated with increases and declines in RSV activity.^28^ Mullins analysed 11 RSV seasons and concluded that there was substantial variability in community respiratory syncytial virus season timing by year and by location.^13^

The last week of RSV epidemic season was frequently in weeks 13‐15 within all methods used in the present study. Complete concordance between ≥1.2% and ≥60% method was found and partial concordance with ≥10% method. Interestingly, according to MEM, most RSV epidemic seasons were shorter (ended earlier). The same has been observed by Vos et al, in the first published study aiming to determine whether the MEM is suitable for use in RSV surveillance using Dutch national virological laboratory surveillance and sentinel GP surveillance data.^19^ The RSV epidemic period was sometimes shorter than the period of low intensity near the end of the epidemic since the post‐epidemic threshold was higher than the low‐intensity threshold.^19^ The present study confirmed the findings of the first study using MEM for RSV surveillance—MEM was simply applied to Slovenian national RSV surveillance data giving an accurate timing of the epidemic season too. Furthermore, MEM provides prospective information on the intensity thresholds of the epidemic and enables preparedness and organisational responses of the healthcare system to react to an increased need during the course of the season of acute respiratory infections. In contrast, the course of the RSV epidemic season can be studied with retrospective methods (such as 1.2% or 60% method) only after the final number of RSV tests performed is known and used as a denominator for the calculation.

A global overview in RSV seasonality showed that, in the majority of countries included in the study, the start, end and peak of RSV activity usually differed by only 1‐3 weeks from season to season, with some countries being the exception to the rule.^28^ Germany presented irregular patterns with early and late start/end of RSV season but a similar duration of seasons^29^—the same pattern we observed in our study. We did not observe any influence of RSV A or B subgroup predominance on the beginning or intensity of the season. This statement needs to be interpreted with caution and represents one of the limitations of the study since the predominant subgroup of the season was determined based on a small number of RSV detections that emerged from the national influenza surveillance system. The impact of RSV subgroup on seasons’ severity remains a controversial issue—some studies showed a clear correlation between RSV subtype and a more severe clinical course of infection.^30^ Earlier and longer seasons were observed in some studies while in others no difference has been found.^15^, ^19^

The start of RSV season is the time to administer RSV specific intervention to the most fragile populations, like prematurely born infants. Yearly shifts in RSV epidemic season onset demand real‐time monitoring of the data, and adapting public health recommendations in case the early season is anticipated. The increasing percentage of RSV‐positive samples might be a simple approach that allows for a timely and reasonable estimation of a developing season and a basis for judicious public health alerts. The ≥3% calculative method could be used as a first alert or early warning that the RSV season might be starting in a short time. We suggest that ≥7% calculative method would be a method of choice for defining RSV epidemic seasons, as the onset/offset and duration are closer to 1.2% and 60% retrospective methods and capture a comparable percentage of annual detections. However, due to changes in the number of tests and positivity rates, in recent years, the proposed calculative method ≥7% should be reviewed after a certain time again to confirm whether it is still suitable for use.

MEM is sophisticated method with a lot of advantages but more practical for every day use are other calculative methods.

## 5 CONCLUSION

In conclusion, the comparison of calculative methods using national data showed good concordance in defining the peak of RSV season with a difference in onset/offset of the epidemic season according to pre‐set thresholds. Despite the comparability to other surveillance data from a temperate climate, even in a small country such as Slovenia, national data of RSV activity should be monitored for public health benefits and response to future challenges that may emerge.

## CONFLICT OF INTERESTS

Dr Grilc E has nothing to disclose.

## AUTHOR CONTRIBUTIONS

**Eva Grilc:** Investigation (lead); Writing‐original draft (equal). **Katarina Prosenc:** Investigation (equal). **Jaro Lajovic:** Formal analysis (lead). **Maja Sočan:** Supervision (lead); Validation (lead).

### Peer Review

The peer review history for this article is available at https://publons.com/publon/10.1111/irv.12779.
